# CRISPR/Cas9 suppression of OsAT10, a rice BAHD acyltransferase, reduces p-coumaric acid incorporation into arabinoxylan without increasing saccharification

**DOI:** 10.3389/fpls.2022.926300

**Published:** 2022-07-22

**Authors:** Svenning R. Möller, Christopher S. Lancefield, Nicola C. Oates, Rachael Simister, Adam Dowle, Leonardo D. Gomez, Simon J. McQueen-Mason

**Affiliations:** ^1^CNAP, Biology Department, University of York, York, United Kingdom; ^2^School of Chemistry and Biomedical Science Research Complex, University of St. Andrews, St.Andrews, United Kingdom; ^3^Biology Department, Bioscience Technology Facility, University of York, York, United Kingdom

**Keywords:** p-coumaric acid, ferulic acid, hydroxycinnamic acid, BAHD acyl transferase, husk, rice, arabinoxylan, cell wall

## Abstract

Ester-linked hydroxycinnamic acids ferulic acid (FA) and para-coumaric acid (*p*-CA) play important roles in crosslinking within cell wall arabinoxylans (AX) and between AX and lignin in grass cell walls. The addition of hydroxycinnamates to AX, is mediated by the Mitchell clade of BAHD acyl-coenzyme A-utilizing transferases. Overexpression of OsAT10 (a Mitchell clade BAHD acyl transferase) in rice, has previously been shown to increase *p*-CA content in AX in leaves and stems, leading to increased cell wall digestibility, potentially associated with a concomitant decrease in FA content. To investigate the physiological role of OsAT10 we established CRISPR/Cas9 rice knock-out mutants devoid of OsAT10. Our analysis of hydroxycinnamic acid content in wild type plants revealed that AX associated *p*-CA is found almost exclusively in rice husks, with very little found in other tissues. Mutant plants were essentially devoid of ester-linked *p*-CA associated with AX, indicating that OsAT10 represents the major enzyme responsible for the addition of *p*-CA to arabinoxylan in rice plants. We found no change in the digestibility of rice husk lacking AX-associated *p*-CA, suggesting that the changes in digestibility seen in OsAT10 overexpressing plants were solely due to compensatory decreases in AX-associated FA.

## Introduction

In plants, cell walls play a multitude of roles from providing support and integrity to cells and tissues to providing an important interface between the plant and its environment. Strength and integrity are key aspects of cell wall functions, but these have to be accompanied by dynamic flexibility to allow growth and development. Cellulose microfibrils composed of paracrystalline assemblages of β1-4, glucans provide the structural framework of the cell wall. Cellulose microfibrils are encased in a matrix of hemicellulose and lignin, and the interactions between these polymers underpins the integrity of the cell wall. The major hemicellulose of graminaceous monocots, such as rice, is a complex arabinoxylan (AX; Scheller and Ulvskov, 2010). AX is made up of a β1-4 xylan backbone, decorated with a range of monosaccharide side chains, predominated by arabinose, but including xylose, galactose and glucuronic acid. Many of the xylosyl backbone residues of AX that lack sugar side chains may be acetylated, and the patterning of xylan decorations impacts the conformation of the hemicellulose and its ability to associate with cellulose (Simmons et al., 2016; Grantham et al., 2017). One of the key features of AX is its ability to form covalent associations between neighboring chains and with the lignin network (Mnich et al., 2020). These linkages primarily occur through ferulic acid (FA) residues, which are present as esters on a subset of the arabinosyl side chains of AX. FA moieties form crosslinks between one another through free radical chemistry to form intra-AX crosslinks (Ralph et al., 1994). The FA sidechains of AX can also form covalent linkages with lignin (Ralph et al., 1992, 1995). Both of these associations play important roles in determining the integrity and digestibility of cereal cell walls (de Oliveira et al., 2015). In addition to FA, the arabinosyl side chains of AX can be ester linked to another hydroxycinnamic acid (HCA), *p*-coumaric acid (*p*-CA), which is not thought to crosslink directly with lignin or neighboring AX chains (Mueller-Harvey et al., 1986; Ishii et al., 1990; Ralph, 2010).

FA and *p*-CA are both also present as esters in the lignin network. The addition of FA and *p*-CA to AX and lignin are believed to be mediated by enzymes belonging to the so-called core Mitchell clade BAHD acyl-CoA transferases (MCBAT), using HCA-CoA as an acyl-donor during biosynthesis (Mitchell et al., 2007; Piston et al., 2010; de Souza et al., 2018, 2019). Using a bioinformatics approach, Mitchell et al. (2007) identified 10 putative MCBAT genes in the rice genome, known as *OsAT1-10.*

Experimental evidence regarding the role of specific MCBATs in different plant species has been produced over the last 10 years since Piston et al. Piston et al. (2010) first demonstrated that simultaneous RNAi knockdown of multiple MCBATs, reduced total FA content in rice. Buanafina et al. (2016) showed that RNAi suppression of BdAT1 (*OsAT1* homolog) in *Brachypodium* resulted in a reduction of total FA content. Furthermore, a T-DNA knockout of *OsAT7* was shown to reduce FA content in rice sheaths (Bartley et al., 2013).

A number of studies have implicated MCBATs in the incorporation of HCAs into lignin. For example, overexpression of *OsAT5* in rice stems led to increased lignin associated ferulic acid (Bartley et al., 2013; Karlen et al., 2016). *OsAT4* has been shown to transfer *p*-CA to lignin *in vitro* (Withers et al., 2012). *OsAT3* homologs in maize (*ZmPMTs*) transfer *p*-CA, FA and to a lesser degree caffeic acid to sinapyl alcohol *in vitro*. However, RNAi knockdown of *ZmPMT* only resulted in a reduction of *p*-CA in cell walls (Marita et al., 2014). In addition, *Brachypodium* knockouts of the *OsAT3* homolog *BdPMT*, completely eliminated sinapyl alcohol bound *p*-CA, while overexpression produced changes to the lignin content and S:G lignin ratio (Petrik et al., 2014). Overexpression of *BdPMT2*, the *OsAT8* homolog, in Arabidopsis resulted in an increase in lignin-associated *p*-CA, similar to *BdPMT1* (Sibout et al., 2016). *BdPMT1* overexpression, in turn, transferred *p*-CA to guaiacyl alcohol, highlighting the broader range of specificities for this enzyme. Taken together, these studies indicate that OsAT3 and 4 (and possibly OsAT8) participate in the addition of *p*-CA to lignin, and OsAT5 in the addition of FA to lignin.

RNAi gene silencing of an *OsAT9* orthologue shows reduced FA-Ara in *Setaria viridis*, and to a lesser degree in *Brachypodium,* indicating it is likely responsible for the addition of FA to arabinosyl side chains in AX. *OsAT9* is also the closest relative to *OsAT10* phylogenetically. These gene silencing studies also demonstrated how changing the content of AX-linked FA has a profound influence on the cell wall and applied plant traits such as digestibility (de Souza et al., 2018, 2019; Li et al., 2018).

OsAT10 overexpression in rice increased incorporation of *p*-CA to AX, and this was accompanied by a decrease in FA on AX, and a concomitant increase in saccharification in stems and leaves (Bartley et al., 2013). A similar effect was seen in switchgrass (Li et al., 2018), suggesting that OsAT10 is responsible for the incorporation of *p*-CA to AX. However, RNAi plants with simultaneous knock downs of *OsAT6* through *10* showed no reduction in *p*-CA in stems and leaves, despite a significant reduction in *OsAT10* transcript (Piston et al., 2010). However, as RNAi can easily reduce the expression of multiple homologous genes, results may have resulted from off-target effect. In addition, RNAi does not fully remove a transcript allowing one to identify leading to potentially ambiguous results. BAHDs may have multiple overlapping specificities (D’Auria, 2006) and overexpression thus may not accurately describe the main target of the enzyme. Using the CRISPR/Cas9 system, targeted cuts can be made to DNA, generating insertions or deletion of base pairs which result in frameshift mutations completely removing the translated protein (Shan et al., 2013). The CRISPR/Cas9 guide only requires 20 base pair to determine the cut and can be designed to minimise potential off-target effects in homologs (Young et al., 2019), unlike RNAi approaches, which typically make use of a much longer hairpin RNA for silencing (Watson et al., 2005).

Mota et al. (2021) recently used *RNAi* gene suppression to reduce the activity of SvBAHD05 (*OsAT1* homolog), a MCBAT in *S. viridis*. Their results showed that suppressing this gene led to a significant reduction in *p*-CA-AX in these plants and this resulted in a modest increase in the digestibility of the affected tissues with commercial cellulases, suggesting that *p*-CA-AX may be a determinant of cell wall recalcitrance to digestion. It has been suggested that *p*-CA can act as a catalyst for lignin formation by radical transfer to sinapyl alcohol (Takahama and Oniki, 1994; Hatfield et al., 2008), but Mota et al. (Currie and Perry, 2007) reported no apparent changes in lignin in their plants with reduced *p*-CA-AX content.

Several plant cell wall components have been suggested to interact with, or bind to, silica (Leroux et al., 2013; He et al., 2015; Guerriero et al., 2018). Several authors have suggested that AX-HCA complexes may act as nucleation sites for silica deposition (He et al., 2013; Soukup et al., 2017). Husks of cereal grain are notably rich in both *p*-CA and silica (Currie and Perry, 2007; Ikram et al., 2017; Gao et al., 2018), suggesting a potential association between these compounds. Lignin content and *p*-CA are also known to increase under silicon deficiency (Goto et al., 2003; Suzuki et al., 2012). These associations between *p*-CA, lignin and silica in the husk underline a possible role in stress defence and grain protection (Boz, 2015).

Whereas most other studies on the Mitchell clade have either utilized RNAi or overexpression, here, we report on the use of CRISPR/Cas9 gene editing to knock out *OsAT10* in rice allowing for full determination of its biological role. Our studies reveal that OsAT10 is responsible for most of the *p*-CA on AX in rice plants. Our studies show that most of the *p*-CA on AX is found in rice husks and that the complete loss of the *p*-CA on AX had no detectable effect on cell wall digestibility or silica content.

## Materials and methods

### Vector construction

Initially, the sgRNA expression cassette was cloned into vector pJIT163-2NLSCas9 (Addgene: Plasmid #53064; Shan et al., 2013), while simultaneously adding a ubiquitin promoter to the *Cas9* gene. The sgRNA expression cassette was made from two fragments with the guide being added as a primer overhang. The primers used for cloning are listed in Supplementary Table S1. A U3 promoter fragment with guide overhang was amplified from UAP1-U3, a sgRNA scaffold fragment with guide overhang was amplified from pICSL90010 and a ubiquitin promoter fragment was amplified from UAP1-OsUbi. pJIT163-2NLSCas9 was opened with Bam-HI and used in a NEBbuilder (New England Biolabs #E2621S) cloning reaction together with the above fragments. The hygromycin cassette was obtained from GoldenGate cloning using vectors pICH47732, pICSL12009, pICL80036 and pICH41421 (Engler et al., 2014; Vazquez-Vilar et al., 2016).

The final *Agrobacterium* expression vectors were cloned by combining the CRISPR/Cas9 expression cassette with the hygromycin expression cassette into the pAGM4723 (Weber et al., 2011) which had been digested with OliI and KpnI, using NEBbuilder. Apart from pJIT163-2NLSCas9 remaining vectors were obtained from MoClo (Weber et al., 2011) and its expansions from (Engler et al., 2014; Vazquez-Vilar et al., 2016).

### Rice transformation

*Agrobacterium* mediated transformation was performed according to (Nishimura et al., 2006). The CRISPR/Cas9 binary vectors were introduced into Japonica rice Nipponbare cultivar. Hygromycin was used as a selection marker and resistant calli were transferred to regeneration media to obtain mature rice plants.

### CRISPR/Cas screening

**Figure 1:**
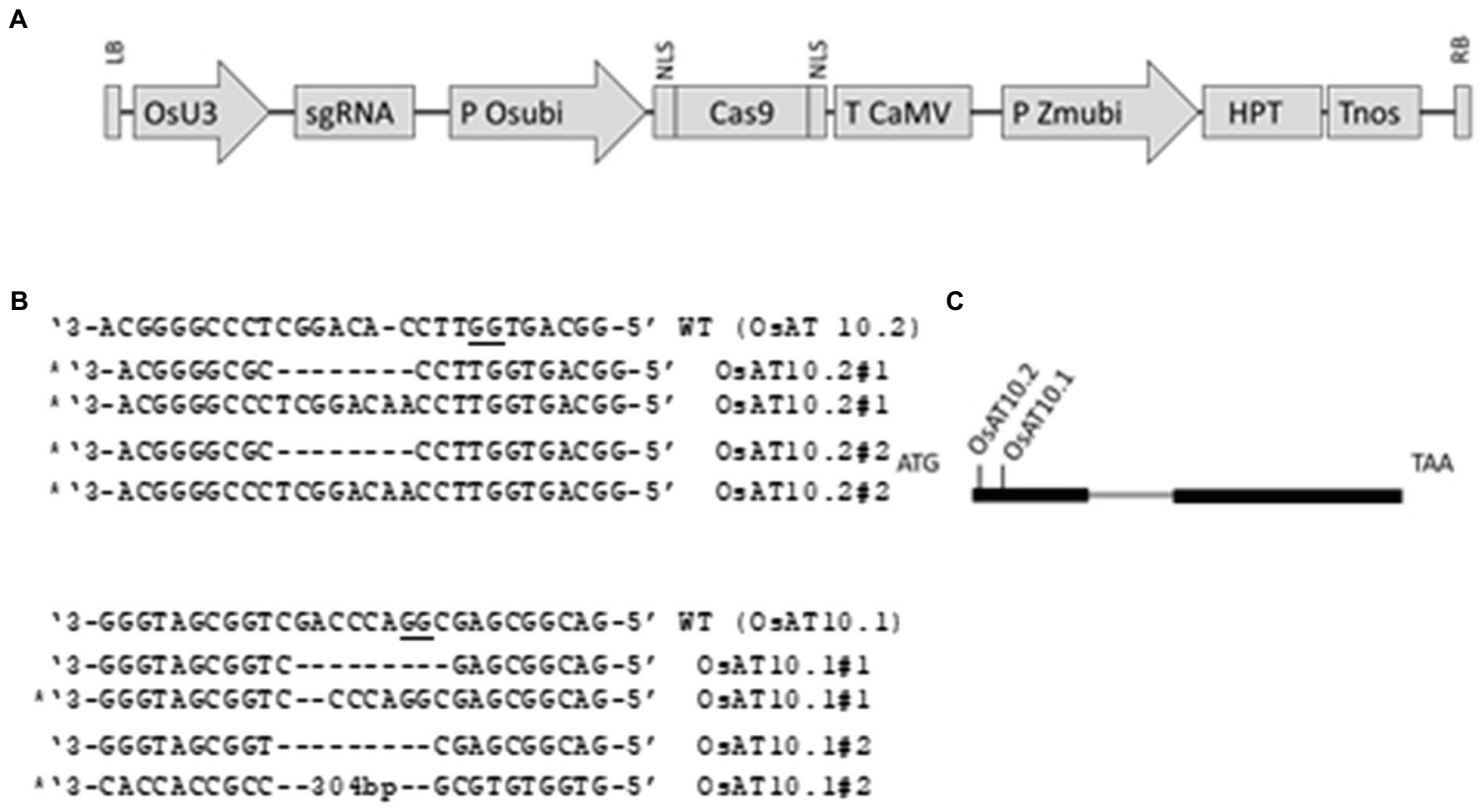
Constructs used and targets for CRISPR/Cas within OsAT10. **(A)** Schematic presentation of the T-DNA structure in the vectors utilized in this study. The expression of the sgRNA scaffold is driven by the rice U3 promoter (OsU3), expression of Cas9 by the rice ubiquitin promoter (P Osubi) and expression of hygromycin selection marker (HPT) by Maize ubiquitin promoter. Abbreviations: NLS, nuclear localization signal; Tnos, Nopaline terminator; T CaMV, Cauliflower Mosaic Virus (CaMV) 35S terminator; LB, Left border; RB, right border. **(B)** Indels present in the original lines, KO lines selected are marked and PAM is underlined. **(C)** Schematic of the OsAT10 gene structure and target site. The intron is marked with gray and the two exons are shown with thick black lines. The location of the two crisprs are marked.

An approximately 600 bp PCR fragment *of OsAT10* (Os06g39390) was obtained from genomic DNA from all the *Osat10* mutants with primers p161 & p162 (see Supplementary Table S1). The fragments were sequenced at GATC-Eurofins with a nested primer p181. Plants with frameshift mutations were propagated further (Figure 1B). Plants were grown in a greenhouse at (30\u00B0C with added lights) T2 plants water was supplemented with 1 ml of Plant Magic Plus Bio-Silicon per litre of watering water. Leaf blades were harvested after 10 weeks and after 16 weeks watering was stopped and fully mature plants were left to senesce as natural, after which the inner dry stems and rice husks were collected. Plant material was freeze dried and powdered using a ball mill. Five T2 or T3 plants of each mutants were analysed as biological replicates in the following experiments. Total plant height was measured every second week.

### Preparation of AIR

Alcohol insoluble residue (AIR) was prepared by adding 2 ml of water to 50 mg of powdered plant material in a 2 ml Eppendorf tube resuspending the powder in the water and shaking by inverting the tube for 30 s before the plant material was pelleted by centrifuging at 12,000RCF for 1 min, the water was removed and replaced with 2 ml of 96% ethanol, followed by two further ethanol washes before the residue was allowed to dry under ambient conditions.

### Silica analysis

Plant material was freeze dried and powdered using a ball mill. Powdered plant material was pressed into disks with hydraulic press and measured using X-ray fluorescence according to (Reidinger et al., 2012).

### Measurement of p-CA and FA by alkaline hydrolysis

Alkaline extraction of p-CA and FA was performed according to (Fry, 2000). Approximately 10 mg of dry plant material was ball milled in a 2 ml tube using metal beads and a ball mill grinder. One milliliter of 1 M NaOH was added and the samples were incubated shaking at 140 rpm at 30°C overnight. The reaction was neutralized by addition of 100 μl 99% TFA and phase extracted twice using butanol. The butanol was evaporated and the residue resuspended in 200 μl MeOH. Samples were analyzed by LC/MS. Separation was carried out on an Acquity I class LC system (Waters United Kingdom, Elstree) using a BEH C18-1.7u 2.1x100mm (Waters), the mobile phases were A) 0.1% acetic acid in H_2_O, and B) 0.1% acetic acid in acetonitrile. The gradient started at 20% B, and increased to 100% B over 3.25 min, where it remained for 0.15 min. The gradient returned to 20% B over 0.05 min, and re-equilibrated over 0.55 min. The total duration of the program was 4 min. The flow rate was 0.5 ml min^−1^, and the injection volume 3 μl. The detector used was a TSQ Endura™ triple quadrupole mass spectrometer (Thermo Fisher Scientific, Altrincham, United Kingdom) with a HESI source, operated in negative MRM mode. Commercially purchased standards of *p*-CA and FA (Sigma) were used to construct standard curves in the range from 12 to 400 μM. The transitions were m/z 163.05 to 119.11, collision energy 15.3 V (*p*-CA) and m/z 193.03 to 134.06, collision energy 16.8 V (FA), with a 0.25 s cycle time, 1.5 mTorr collision gas pressure, ion transfer tube temperature of 333°C, vaporizer temperature of 317° C and a spray voltage of 3.5 kV. Q1 and Q2 resolutions were 1.2 Da. Data processing was performed using Thermo Xcalibur 4.0.27.10 QualBrowser and QuanBrowser software.

### Measurement of p-CA-arabinose and FA-arabinose by mild acidolysis

Measurement of *p*-CA-arabinose (*p*-CA-ara) and FA-arabinose (FA-ara) was performed essentially according to (Lapierre et al., 2018). One millilitre of acidolysis reagent (dioxane, methanol and aq 2 M HCl 60:30:10, v/v/v) was added to 5–10 mg of AIR in a screwcap Eppendorf vial. The vials were incubated for 3 h at 80°C in an Eppendorf tube shaking at 1200 rpm. After cooling, 2 ml of water and 20 μl of 0.1 μg/μl *o*-coumaric acid in methanol was added. *o*-coumaric acid was used to correct for loss during the phase extraction, although this was minor. The diluted mixture was extracted with EtOAc. EtOAc was evaporated and the pellet dissolved in 150 μl of MeOH, which was analysed by HPLC using UV detection. 10 μl of sample was injected onto an XBridge BEH Shield RP18 Column, 130 Å, 3.5 μm, 1 mm × 100 mm (Waters Part Number: 186003149) using 1.23 ml/min flowrate and a binary gradient made up of 0.1% acetic acid and methanol. 0–60 min going from 5 to 65% MeOH, followed by a rinse 60–65 min from 65 to 90% methanol and then 65–70 min back to 5% methanol or 0–30 min going from 5 to 70% MeOH, followed by a rinse 30–35 min from 70 to 90% methanol and then 35–40 min back to 5% methanol. The eluent was monitored using a photodiode array detector and HCA and HCA-conjugates were determined by absorption at 320 nm. Data processing was performed using Waters Empower Pro software. As an external standard we used a series of five dilution of *p*-CA and FA spanning 0.12 μg/μl and 0.14 μg/μl to 0.0075 μg/μl and 0.0088 μg/μl, respectively. Putative *p*-CA-ara and FA-ara peaks were extracted, evaporated, and resuspended in 100 μl MeOH to subjected to identification using MALDI-TOF/TOF. Acquisition was performed using a Bruker Ultraflex III equipped with a Nd:YAG smart beam laser with positive ionisation in reflectron mode post spotting samples 1:1 with 20 mg/ml 2,5-dihydroxybenzoic acid (DHB) matrix. MS^1^ precursor ions unique to the samples and absent in a DHB blank, were manually selected for MS/MS fragmentation performed in LIFT mode without the introduction of a collision gas. Bruker flex Analysis software (version 3.3) was used to perform spectral processing and peak list generation. MS2 product ion spectra were manually inspected and annotated for analyte identification.

### Monosaccharide analysis

Non-cellulosic monosaccharide analysis was performed using high-performance anion exchange chromatography (HPAEC; Carbopac PA-10; Dionex). Water extractable arabinoxylan was fractionated from approximately 40 mg of dry powdered plant material with 1 ml of water overnight. The extract was filtered through a 0.45 μm polytetrafluoroethylene (PTFE) filters and evaporated to obtain a pellet. Three milligram of AIR or the pellet from water extractable arabinoxylans were hydrolysed with 0.5 ml 2 M trifluoroacetic acid (TFA) for 4 h at 100°C, cooled to room temperature and evaporated completely. The pellet was resuspended in 200 μl of pure water, filtered through a 0.45 μm PTFE filter and separated by HPAEC as described in Jones et al. (2003). The monosaccharides were quantified by using an external calibration containing seven monosaccharide standards at 0.3, 0.6, and 1 μM (arabinose, fucose, galactose, glucose, mannose, rhamnose, and xylose), that were subjected to acid hydrolysis in parallel with the samples.

### Saccharification assays

Powdered AIR was loaded into 96-well plates, using a custom-made robotic platform (Labman Automation, Stokesley, North Yorkshire, United Kingdom), and saccharification assays were performed according to Gomez et al. (2010) after water pretreatment. Enzymatic hydrolysis was carried out using an enzyme cocktail with a 4:1 ratio of Celluclast and Novozyme 188.

### Nuclear magnetic resonance material preparation

Rice plant samples were prepared based on previously published methods (Kim and Ralph, 2010; Mansfield et al., 2012). Dried samples (*ca.* 600 mg) were finely milled using a Planetary Fritsch Pulverisette 7 Micro Mill spinning at 600 rpm with nylon vessels (45 ml) containing ZrO_2_ ball bearings (14 mm × 2, 10 mm × 9). The total grinding time was 1 h 55 min consisting of 15 min intervals with 5 min interval breaks to avoid excessive heating. To produce lignin enriched fractions ball milled samples were treated with Cellic^®^ CTec2 enzyme cocktail (Novozymes, Denmark, activity 120 FPU/ml) which contains a mixture of plant cell wall degrading enzymes. The milled samples (380 mg) were suspended in 50 mM sodium acetate buffer (10 ml, pH 5.5) and the Cellic^®^ CTec2 enzyme cocktail was added (126 μl, 40 FPU/g substrate). The mixture was incubated on a rotary shaker at 50°C for 5 days. The insoluble residue was collected by centrifugation (4,000 rpm, 2 min), washed twice with water and then freeze dried to give fine powders. Yield from WT rice husk was 151 mg (40%) and from the *Osat10* mutant 141 mg (37%). Lignin enriched fractions were transferred directly to a 5 mm Nuclear Magnetic Resonance (NMR) tube (75 mg) and roughly distributed up the sides of the tube. Pre-mixed DMSO-d_6_/pyridine-d_5_ (4,1) was directly added into the NMR tube and vigorously shaken for a few seconds to mix the contents before being placed in a sonicator bath for 1 h.

### NMR acquisition and processing

NMR spectra were acquired on a 600 MHz Bruker Avance III HD 600 MHz spectrometer with TCI cryoprobe. 2D ^1^H–^13^C HSQC spectra were acquired using a standard Bruker pulse program (hsqcetgpsisp2.2). The NMR spectra were acquired from 12 to −2 ppm in F2 (1H) using 2048 data points for an acquisition time (AQ) of 122 ms, an interscan delay (D1) of 1 s, 47–137 ppm in F1 (^13^C) using 144 increments of 16 scans. The spectrum was processed using squared cosine bell in both dimensions and LPfc linear prediction (18 coefficients) in F1. Interactive integrations of contours in 2D HSQC plots were carried out using Bruker’s TopSpin 3.6 (Windows) software, as was all data processing. Semi-quantitative analysis of the lignin components was accomplished by integration and comparison of the S2/6 and G2 resonances in the aromatic region and the signals for each structural unit in the oxygenated alkyl region. Abundances of the lignin linkages are expressed as the number of linkages per 100 C_9_ units. Relative amounts of *p*-CA and FA are expressed as a simple percentage relative to the lignin aromatics. Assignments of NMR spectra are made by comparison to previously published data (Kim et al., 2008; Mansfield et al., 2012; Lam et al., 2017, 2019; Takeda et al., 2019).

## Results

### Generating CRISPR/Cas knockout mutants

**Figure 2:**
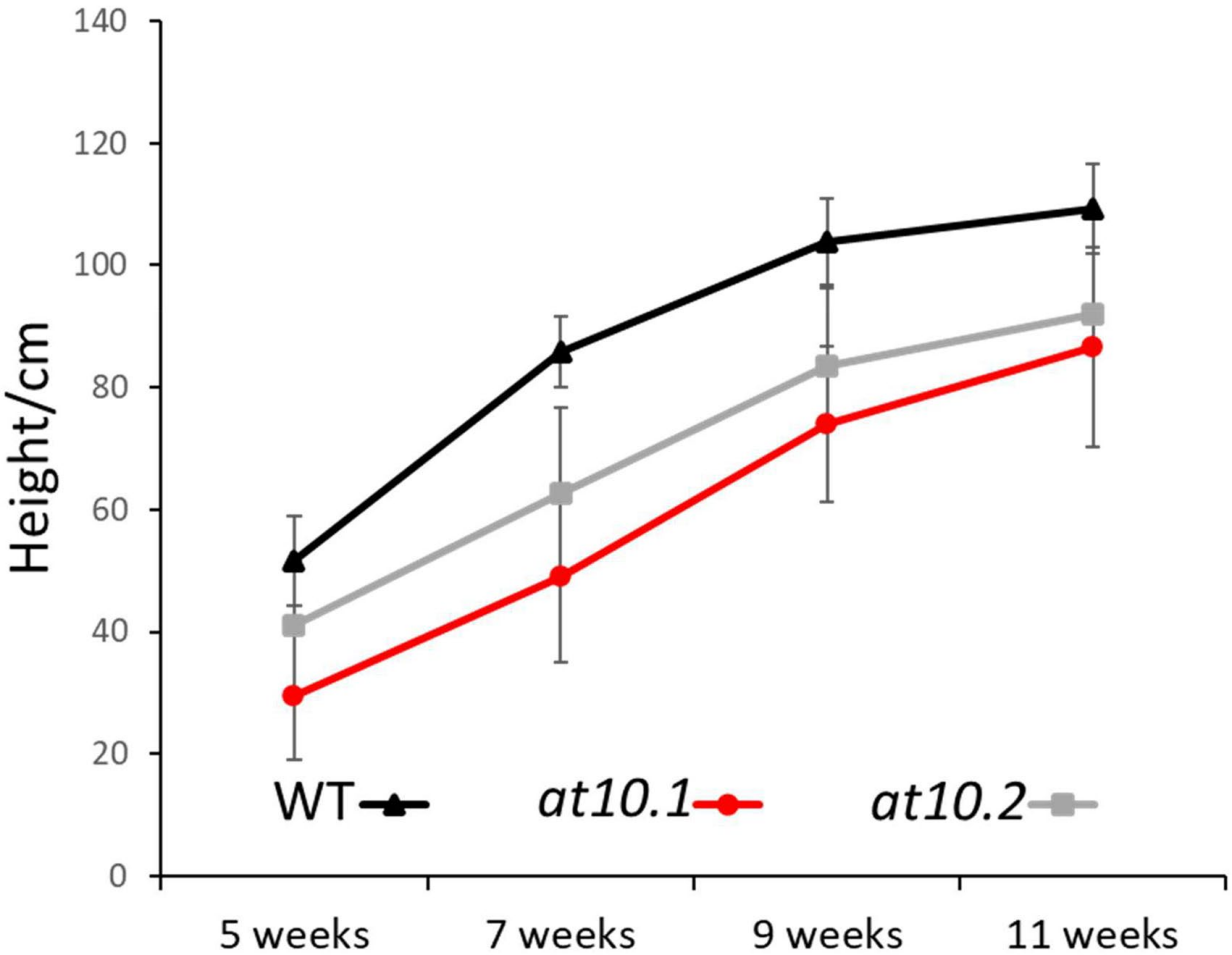
Growth of rice plants studied. Total height was measured at different stages of growth. Wild Type (Black), OsAT10.1 (Gray) and OsAT10.1 (Red). Error bars represent standard deviation.

We designed two RNA guide sequences (OsAT10.1 and OsAT10.2; Figure 1) using sgRNA2.0 scorer (Chari et al., 2015, 2017) while taking into account the potential off targets and sites with restriction enzymes using CRISPRdirect (Kawahara et al., 2013; Naito et al., 2015). Multiple T1 mutants for both guides were obtained. From these two transformations with biallelic mutations we selected two as independent lines for each guide RNA. By having two knockouts made using different guide RNAs we greatly reduced the possibility of the observed phenotypes arising from off-target effects. We identified homozygous T2 plants with frame-shift mutations preventing the protein translation by sequencing PCR products generated across the target sites. The mutant plants did not show any obvious visible phenotype. However, when we measured height in third-generation mutants grown under greenhouse conditions they grew slightly slower than the corresponding wild type plants with *p* < 0.05 *via* Student’s *t*-test Figure 2.

### Osat10 mutants lack AX-associated p-CA, which is mostly found in the husks of wild type plants

**Figure 3:**
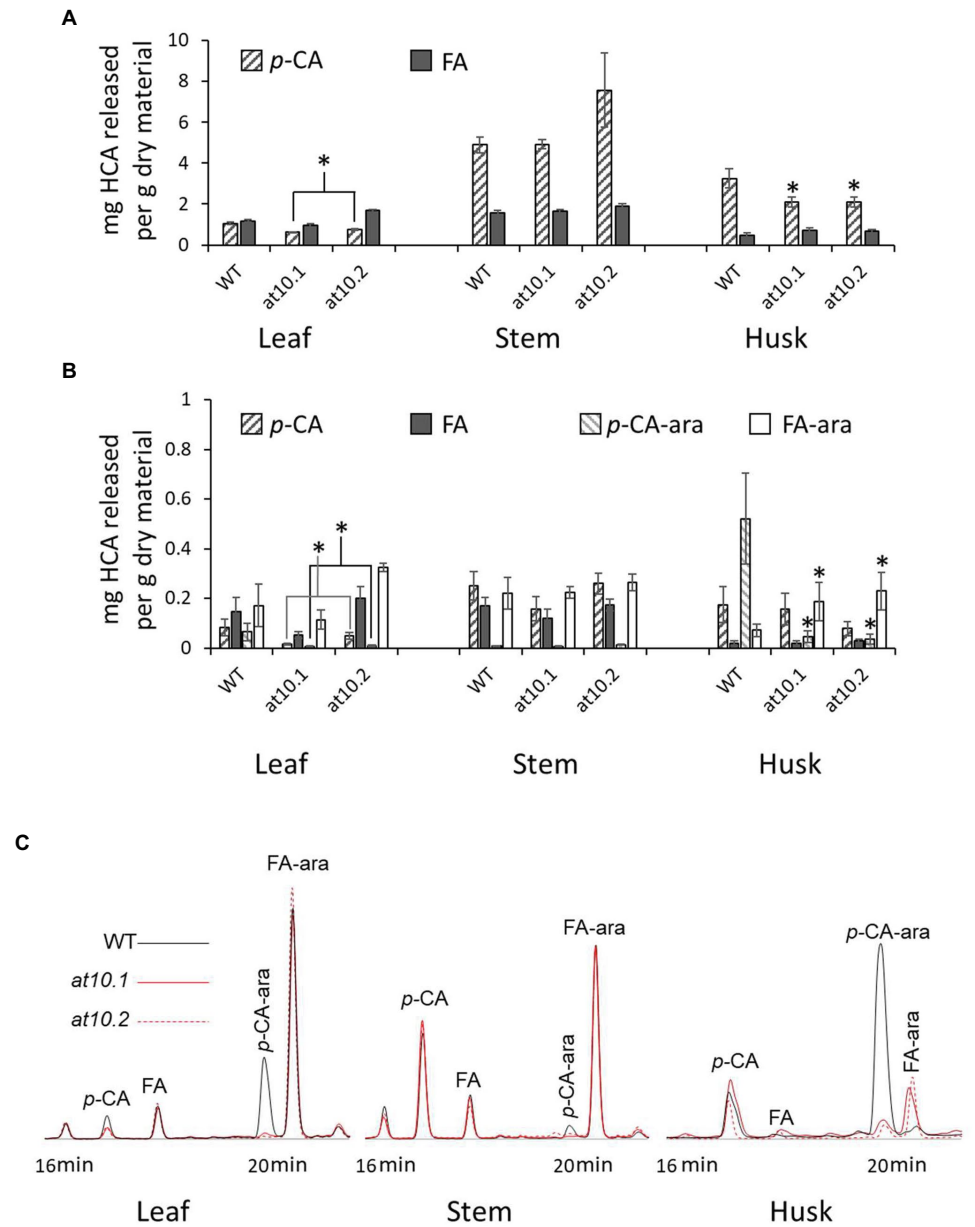
Hydroxycinnamic acids contents and identification in *Osat10*. **(A)** Determination of total ester-linked ferulic acid (Grey bars) and *p*-coumaric acid (Hatched bars) content of stems, leaves and husks as obtained from alkaline hydrolysis per gram of dry material. **(B)** HPLC-UV determination of *p*-CA (Hatched bars), FA (Grey bars), *p*-CA-ara (Hatched bars, lighter) and FA-ara (White bars) released by mild acidolysis of rice leaves, stems and husks from *OsAT10.1, OsAT10.2* and WT. Error bars represent standard deviation. *Significant difference at *p* < 0.05 *via* Student’s t test. **(C)** HPLC chromatograms from leaves, stems and husks, showing UV absorption at 320 nm. Wild type is black OsAT10.1 is red and OsAT10.2 is dotted. Compounds: *p*-CA-ara; methyl 5-O-E-*p*-coumaroyl-L-arabinofuranoside, FA-ara; methyl 5-O-E-feruloyl-L-arabinofuranoside, FA; Ferulic acid, *p*-CA; *p*-Coumaric Acid.

We initially measured total *p*-CA and FA content in rice stems, leaves and husks, using alkaline hydrolysis followed by analysis by LC/MS in 10–16 weeks old T2 rice mutants and wild type plants (Figure 3A). This procedure releases all exclusively ester-linked HCAs from both AX and lignin. In stems there were no discernible differences between wild type and the *Osat10* knockout mutants in terms of total ester-linked HCA content. However, there was a significant reduction in ester-linked *p*-CA content in the mutant husks and a smaller reduction was apparent in mutant leaves compared to wild type. This is compatible with published expression data for *OsAT10* at RiceXPro (Sato et al., 2011, 2013; Supplementary Figure S4) that suggests *OsAT10* is almost exclusively expressed in husks. While leaf expression appears nearly absent this is agreed by data presented by Piston et al. (2010) and RNAseq data from the Rice Genome Annotation Project (Ouyang et al., 2007).

Because ester-linked *p*-CA can be associated with both AX and lignin we used the method of Lapierre et al. (2018) to extract and measure HCAs and their arabinose conjugates in order to distinguish the AX associated HCAs as their arabinose conjugates from those associated with lignin (Figure 3B). This method provides a measure of the relative amount of *p*-CA and FA linked to arabinose, as well as *p*-CA and FA. Although, this amount only contains a partial contribution from arabinose bound *p*-*CA.* Using HPLC methods, we were able to identify the *p*-CA and FA peaks using standards, as well as the peaks of methylated *p*-CA and FA by running the standards through the acidolysis process. While we did not have the 5-O-*p*-coumaroyl- and 5-O-feruloyl-L-arabinofuranoside standards, the two peaks at 32.9 or 20.1 min and 34.3 or 20.6 min have similar UV–vis spectra to the *p*-CA and FA, respectively. To confirm the identification of these peaks, we subjected them to the mild alkaline hydrolysis treatment and re-analysed them, yielding peaks corresponding to *p*-CA and FA, respectively. Further confirmation of the peak identities was obtained using MALDI-TOF/TOF, confirming their identity as *p*-CA-Ara and FA-Ara derivatives (Supplementary Figure S1).

In wild type husks, we found that the ratio of *p*-CA-ara to FA-ara was about 6:1, making *p*-CA likely the major HCA found in AX in rice husks. In contrast, the *Osat10* knockout mutants were almost completely devoid of *p*-CA-ara (Figures 3B,C). This was corroborated by analysis of multiple rice tissues revealing that the mutants lack *p*-CA-ara in all major tissues. Our analyses reveal that *p*-CA-ara is a minor component in wild type rice leaves, almost absent in wild type stems, and essentially absent in all tissues of the *Osat10* mutants (Figures 3B,C). The *Osat10* mutants exhibit a compensatory increase in FA content of AX in rice husk mutants with roughly twice the amount seen in wild type husks (Figure 3B). There is, however, a small peak remaining at the *p*-CA-ara peak in the *Osat10* mutant husks.

**Figure 4:**
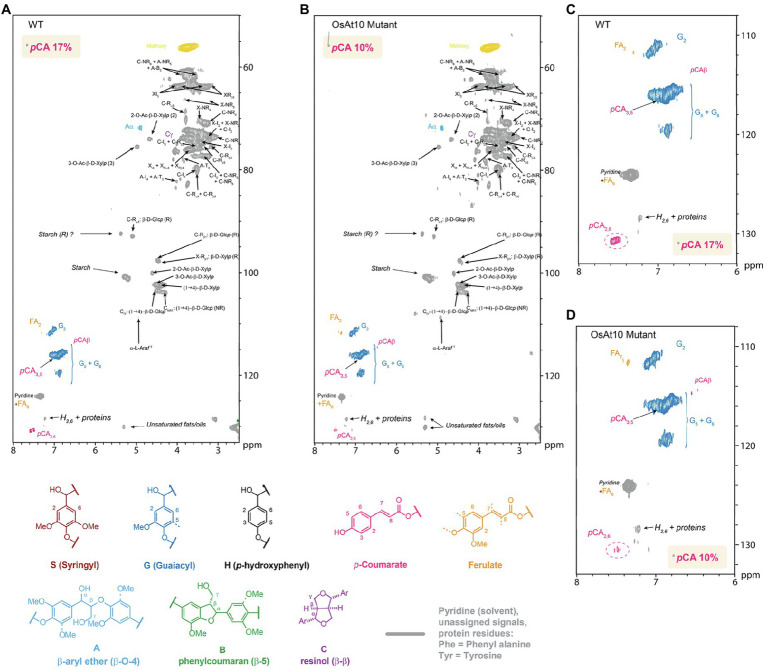
Whole cell gel-state 2D-NMR heteronuclear single-quantum coherence (HSQC) analysis of rice husk cell walls from **(A–C)** the WT control and **(B–D)** OsAt10 knockout mutant. The analytical data are from volume integrals of correlation peaks representing reasonably well-resolved C/H pairs in similar environments; thus, they are from G_2_ and p-CA_2/6_ in the aromatic region, with corrections applied for units that have two C/H pairs per unit. The p-CA_2/6_ abundances are reported on a per 100 C9 unit basis relative to G = 100 C9 units. The small amounts of S units present in the samples were too low to be accurately quantified in these cases.

It has been suggested that *p*-CA might aid in the nucleation of lignin by performing radical transfer to sinapyl alcohol (Takahama and Oniki, 1994). To determine if there were any related effects on lignin in rice husks of the mutant plants, we initially used gel-state 2D-HSQC NMR to examine the cell wall composition. Gel-state 2D-NMR was performed according to (Kim and Ralph, 2010; Mansfield et al., 2012) to measure the aromatic units. We observed a 40% decrease in *p*-CA in the knockout mutant, in good agreement with the changes observed by alkaline hydrolysis (Figure 4). To explore if these changes were associated with the lignin or AX fractions, we treated ball milled rice husk with a biomass degrading enzyme cocktail (Cellic CTec2) to create a lignin enriched residue, depleted in cellulose and hemicelluloses which was also subjected to 2D-HSQC NMR analysis. In contrast to the non-digested husks, no change in *p*-CA was apparent in the lignin enriched residue from the *Osat10* mutants (Supplementary Figure S3). These results confirm the almost complete loss *p*-CA in the AX fraction of husk cell walls in the mutant plants, with no associated discernible changes in lignin composition.

### Loss of AX p-Coumaroylation of AX is not accompanied by alterations in the matrix monosaccharide composition, silica content, or cell wall digestibility in rice husk

**Figure 5:**
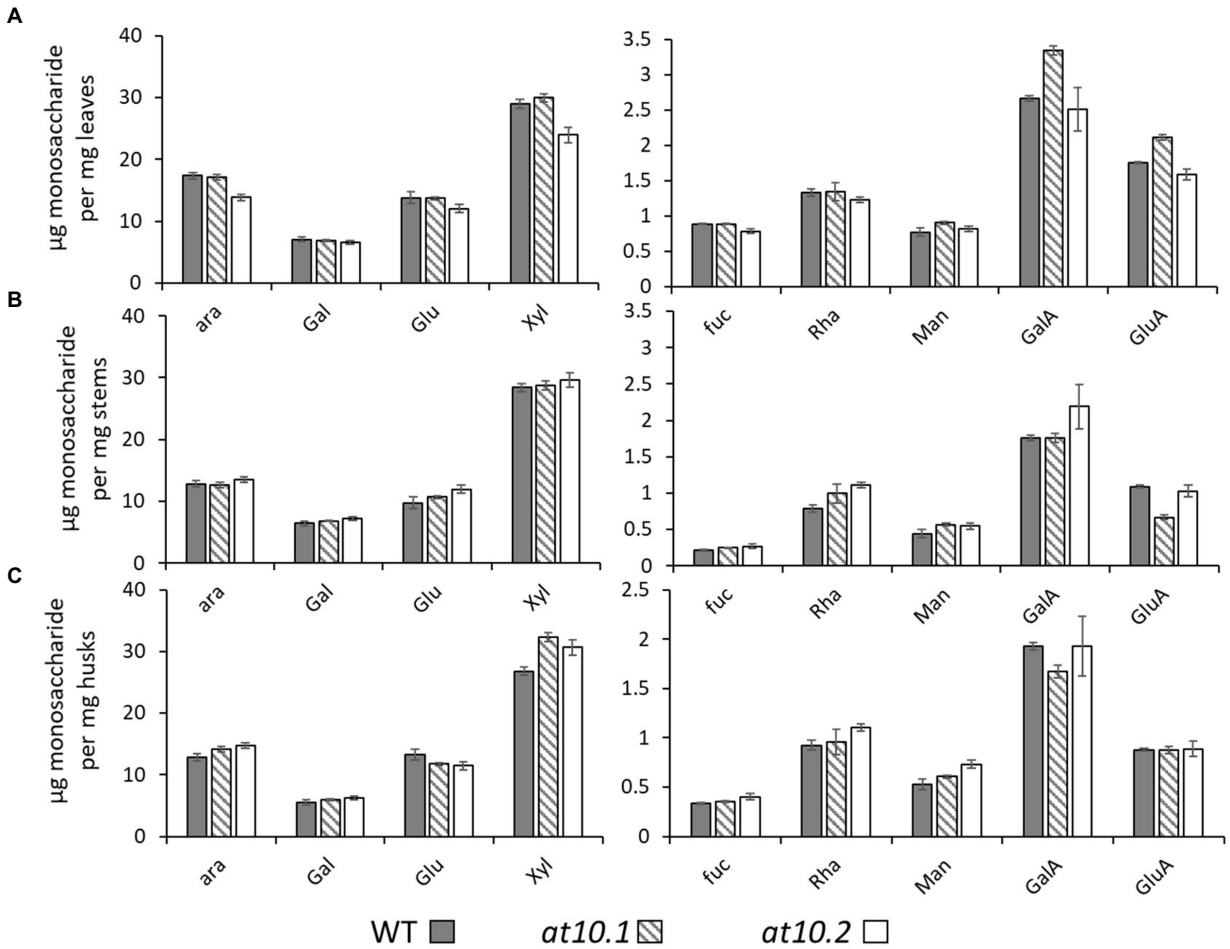
Monomeric sugar analysis. μg of Monomeric sugars pr. mg dry material released by TFA. Material was taken from leaves **(A)**, stems **(B)** and husks **(C)**. WT is shown as Gray bars, OsAT10.1 Hatched bars and OsAT10.2 White bars. Error bars represent standard deviation. Student’s t-test was run between WT and both mutants to check for significance.

**Figure 6:**
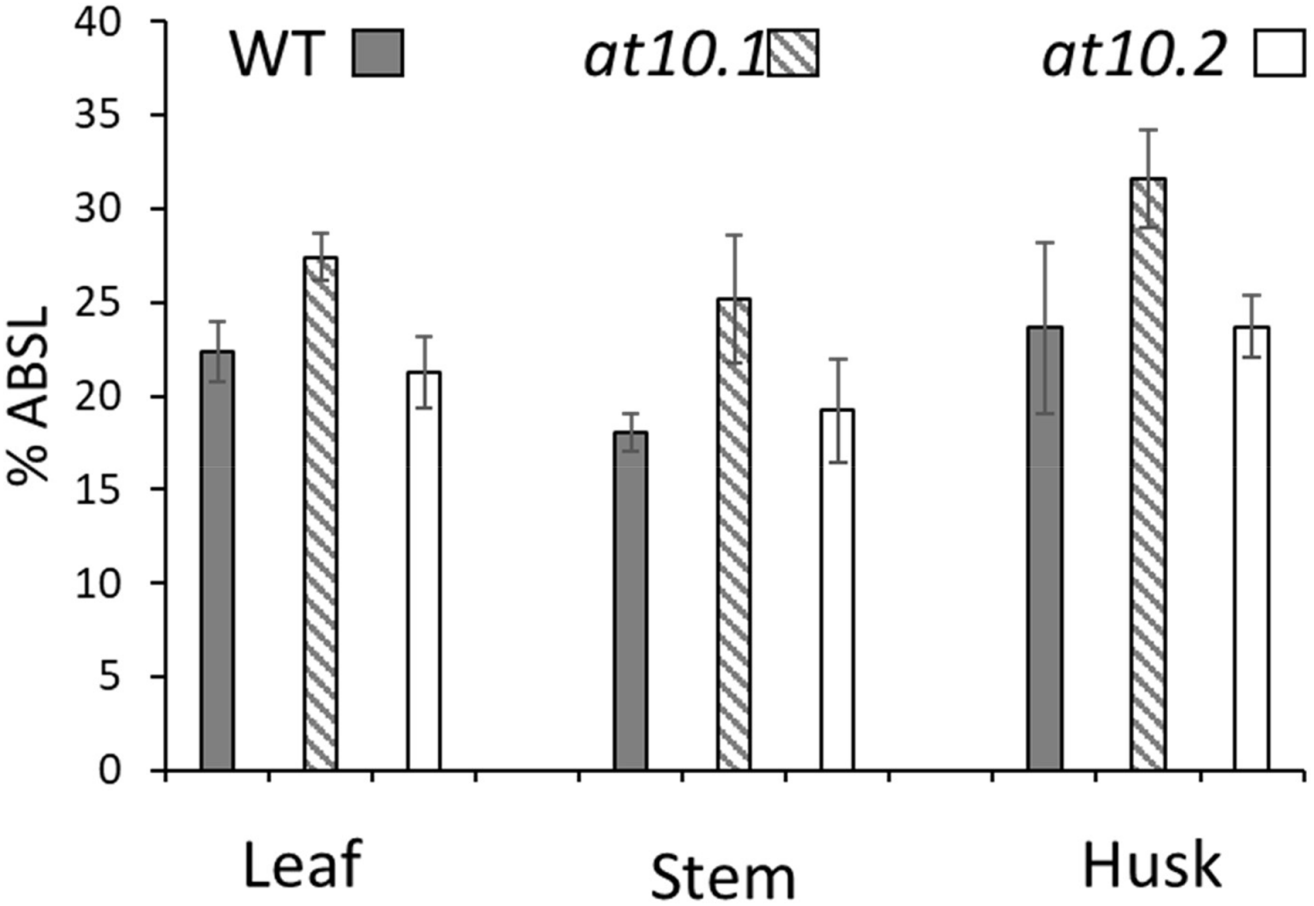
Acetyl bromide soluble lignin analysis. The lignin content of WT (Gray bars), Osat10.1 (Hatched bars), Osat10.2 (White bars) as mass % acetyl bromide soluble lignin. The bars indicate the standard errors. Student’s t-test was run between WT and both mutants to check for significance.

We sought to establish if the loss of *p*-CA on AX had any impact on the composition or enzyme digestibility of rice husk cell walls. To analyze the monosaccharide composition of cell wall polymers, we treated AIR obtained from rice husks stems and leaves with TFA to hydrolyze non-crystalline polysaccharides, and analyzed the resulting monosaccharides by HPAEC. We observed no significant changes in the monosaccharide composition in the *Osat10* mutants compared to those from wild type (Figure 5). We also investigated whether the loss of *p*-CA in husk cell walls, led to any alterations in the extractability of wall polysaccharides, by changing the chemical properties of AX. To tests this, we subjected rice husks and leaves to hot water extraction and quantified the sugars extracted after a TFA treatment similar to the one applied to AIR samples (Supplementary Figure S2). No significant differences in water extractability of polysaccharides were observed between mutant and wild type. To measure total lignin content, we treated AIR from leaves, husks and stems with acetyl bromide in glacial acetic acid in order to solubilize the lignin, after which lignin content was determined by absorption at 280 nm (Figure 6). As with wall monosaccharide composition, no significant difference was observed between the wild type and both mutants.

**Figure 7:**
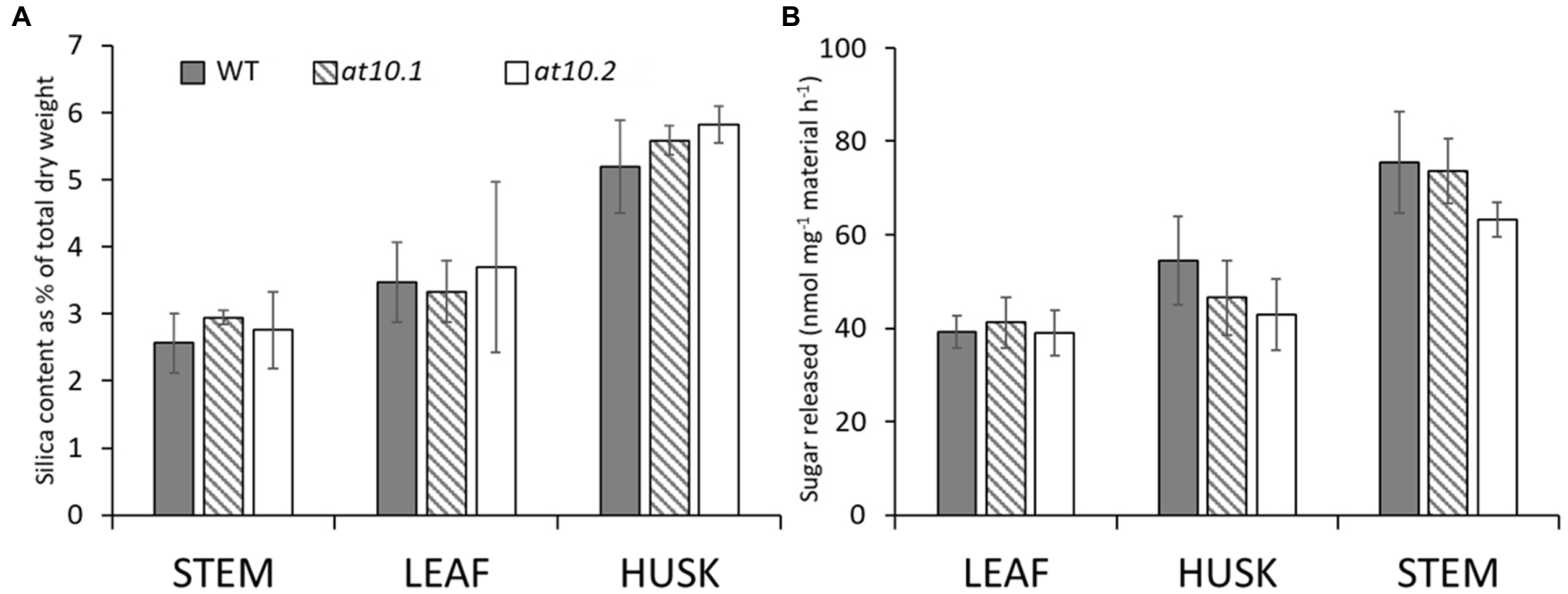
Silica and saccharification analysis. **(A)** Analysis of silica content measured by X-ray fluorescence and **(B)** saccharification analysis of leaves, husks and stems from wild type (grey bars) and OsAT10 mutant OsAT10.1 (Hatched bars) OsAT10.2 (Empty bars) The ground material for saccharification analysis was digested with a commercial cellulase for 8 h at 50°C following a mild water pre-treatment. Student’s t-test was run between WT and both mutants to check for significance.

Bartley et al. (2013) showed that overexpression of *OsAT10* increases *p*-CA levels in rice stem AX, with a concomitant decrease in FA and that this led to increased enzymatic digestibility on the straw. We investigated whether the lack of *p*-CA substitutions on AX in rice husks in the *Osat10* mutants resulted in any changes in enzymatic digestibility. These experiments used an automated platform to perform enzymatic saccharification assays on dried biomass powder using a commercial cellulase cocktail (Gomez et al., 2010). We did not observe any significant differences between the digestibility of mutant and wild type husks, leaves or stems in these experiments (Figure 7A).

The husks (lemma) of rice and other cereal seeds play a protective role and are notable for having high contents of silica and *p*-CA (Hunt et al., 1984; Gao et al., 2018), and connections between aromatic cell wall components and silica have been suggested by some authors (He et al., 2015; Zhang et al., 2015; Soukup et al., 2017). We measured the levels of Si in rice husks from mutant and wild type plants, but found no significant difference between the two (Figure 7B).

## Discussion

MCBATs are responsible for the incorporation of HCAs into plant cell walls, including *p*-CA and FA into lignin and into AX. The roles of specific MCBAT genes are starting to be defined, but off-target effects in RNAi studies, and reliance on the analysis of total *p*-CA and FA content (rather than lignin- and AX-associated HCAs) has led to ambiguities regarding specific roles. Previous studies implicated OsAT10 as being responsible for *p*-CA incorporation into AX (Bartley et al., 2013; Li et al., 2018). However, these studies involved overexpression of the OsAT10 gene resulting in increased AX- *p*-CA content in rice and switchgrass, but did not demonstrate the role of the gene in normal plants. We used a gene knockout approach to study the role of OsAT10 in rice, and examined the levels of *p*-CA and FA in both lignin and AX to allow unambiguous assignment of the role of OsAT10 in rice.

The observation that lowering AX bound FA in grass cell walls decrease biomass recalcitrance (de Souza et al., 2018, 2019) has increased the interest to understand the role of each family member of the BAHD acyltransferase clade of genes. In rice, overexpression and suppression have shed light upon the role of some members of this gene family. Indeed, the roles of *OsAT5*, *OsAT7, OsAT4,* and *OsAT10* have been studied and their roles partially established. Bartley et al. (Bartley et al., 2013) studied the role of *OsAT10* using a promoter tag overexpression system and showed that the overexpression of this gene increased the incorporation of *p*-CA to AX, reduced the amount of FA linked to AX and decreased the recalcitrance of the biomass. In the present work we used a CRISPR/Cas9 approach to generate lack-of-function genes for *OsAT10* allowing for detailed analysis if the gene function without the possible side effects of gene overexpression.

### OsAT10 is likely singularly responsible for the addition of p-CA to GAX in rice

Our experiments allow an unambiguous appraisal of the role of the OsAT10 in rice plants. In our mutants, *p*-CA-ara appears to be almost completely absent in all tissues examined, indicating that this gene is responsible for most, or all, of the addition of *p*-CA to AX in Nipponbare rice. By quantifying the total *p*-CA and the *p*-CA esterified to arabinose, we show that OsAT10 is not responsible for the addition of *p*-CA to rice lignin. Analysis of the total *p*-CA in rice husks indicates a decrease within the same range that we find using a 2D NMR approach. However, once the arabinoxylan has been removed by Cellic Ctec2 treatment to create a lignin enriched fraction we do not see changes in lignin associated *p*-CA between wild type and mutants. This further demonstrates *OsAT10* function is likely to exclusively transfer *p*-CA onto arabinose for incorporation into AX.

The synthesis of AX occurs in the Golgi, and a cytosolic localisation of BADH activity would be required for the acylated product to be transported into the Golgi (Myton and Fry, 1994; Fry, 2004). UDP-arabinofuranose is synthesised in the cytosol and has therefore been proposed as the substrate for both OsAT10 and OsAT9 (Buanafina, 2009; Rautengarten et al., 2011; Seifert, 2018), requiring a glycosyltransferase to incorporate the product onto AX in the Golgi. The lack of subcellular location signal peptide in *OsAT10* indicating a cytosolic localization, is coherent with the proposed biosynthetic pathway and de Souza et al. (de Souza et al., 2018) claims the *in-situ* localization green fluorescence protein fusion with the wheat ortholog is cytoplasmic. The BAHD enzymes have been found to be cytoplasmic (D’Auria, 2006). The role of OsAT10 MCBAT is underpinned by having close homologs only in the commelinids and the presence of AX conjugated *p*-CA only in grasses (Li et al., 2018). Although data presented by (Piston et al., 2010) indicates that *OsAT10* is significantly expressed in stems, the Rice Xpro database (Sato et al., 2011, 2013) suggests that *OsAT10* is mainly expressed in rice husks. In the present work, we found minimal amounts of *p*-CA-ara in stems of wild type plants. Interestingly, we observed an increase in FA-ara in our *Osat10* mutant husks. (de Souza et al., 2018, 2019) reported an increase in *p*-CA-ara upon down regulation of *OsAT9* homologs in *Setaria* that add FA to AX. It seems possible that, *OsAT10* and *OsAT9* compete for the available UDP sugar substrate and/or accessible arabinose on AX in stems, and when either *OsAt10* or *OsAT9* are downregulated, one activity takes prevalence over the other.

### Loss of p-CA-AX does not affect cell wall digestibility

*Osat10* mutants do not show any change in saccharification with a commercial cellulase cocktail. This supports the hypothesis that the increased cell wall digestibility seen in OsAT10 overexpressor lines (Bartley et al., 2013; Li et al., 2018) was caused by a reduction in the amount of FA in AX, a side effect of the gene overexpression. The loss of *p*-CA-ara in the OsAT10 mutants also had no impact on the digestibility of the biomass in leaves, stems or husks. Our results contrast with those recently published by Mota et al. (Mota et al., 2021), which showed that gene suppression of *SvBAHD05* in *S. viridis*, resulted in decreased levels of *p*-CA-AX in most tissues, and that this was accompanied by increased biomass digestibility. While species differences might account for this apparent contradiction, we note that the gene suppression experiments in *S. viridis* only resulted in partial suppression of the expression of *SvBAHD05* and this was accompanied by only partial reduction in the amount of *p*-CA-AX in the tissues, compared to our gene knockout experiments with almost complete loss of *p*-CA-AX. A significant reduction in *p-*CA-AX was also seen in the rice xylosyltransferase mutant *xax1* (Chiniquy et al., 2012) indicative of how other enzymes may influence *p*-CA-AX content. In addition, some small reductions in the amount of FA-AX are also evident in the work published by Mota et al. (Mota et al., 2021), which may be due to off-target effects on other MCBATs in *S. viridis,* or to promiscuous activity of the encoded enzyme. It seems possible that the small increases in biomass digestibility seen in these experiments in *S. viridis*, may be the result of the slight reduction in FA-AX that they observed rather than due to the reduction in *p*-CA-AX. In contrast, our work in rice resulted in complete loss of function of the *OsAT10* gene, resulting in almost complete loss of *p*-CA-AX, and no significant change in biomass digestibility. We may observe a small decrease in the growth of the mutant plants. We are not sure if this is indeed due to the plant compensating for the loss of OsAT10 or a result of the plant experiencing a fitness loss from being transformed with Cas9.

### OsAt10 knock out mutants show no changes in lignin composition and silica content

Our results show that *p*-CA is a very minor component of AX in most rice tissues, with the exception of rice husks. Rice husks provide a protective layer to the seed and these tissues are notably rich in *p*-CA-ara and silica. The loss of *p*-CA-AX in the *Osat10* mutant rice husk had no impact on silica content and lignin composition, indicating no notable interactions between these wall components. (Hatfield et al., 2008) studied the possible role of *p*-CA as an electron donor in lignin polymerisation but found no effect of free *p*-CA *in vitro*. This lack of effect could be due to either the low AX conjugated *p*-CA concentration or to the low mobility of *p*-CA within the cell wall to contribute as an electron donor. However, other compounds, such as coniferyl alcohol, have been shown to enhance sinapyl alcohol oxidation (Takahama and Oniki, 1997). Our measurements also show that while FA is the predominant AX modification in most rice tissues, *p*-CA is the major HCA in AX in husks. This is in agreement with observations in barley glumes, which can be considered anatomically similar to rice husks, and also have *p-*CA as the predominant HCA in AX (Lapierre et al., 2018).

The silica content and the silica cell morphology (Data not shown) in stem and husks was not changed by the loss of *p*-CA-ara. Several other cell wall compounds have been implicated in silica deposition including, e.g., callose (Guerriero et al., 2018), homogalacturonan (Leroux et al., 2013) and hemicellulose (He et al., 2015). Although it is possible that the total amount of silica is mostly driven by the uptake and not dependant on a binding partner, a recent study has proposed a specialized protein (Kumar et al., 2021) as being responsible for silica precipitation in sorghum silica cells.

Although we did not examine the other potential physiological roles for AX associated *p*-CA, there is abundant evidence that they are a key component of abiotic and biotic stress responses. They have been implicated as fungal pathogens and insect deterrents (Santiago et al., 2007, 2008; Buanafina, 2009; Lanoue et al., 2010) and as UV-protectant and free radical quenchers (Ruhland et al., 2005; Stelzner et al., 2019). The higher content of AX conjugate *p*-CA in husks, pollen, and fruits, relative to other tissues supports their protective role in plant organs that play an essential role in reproduction.

In summary, our work shows that OsAT10 is responsible for AX coumaroylation in rice and may be the only gene conferring this activity in aerial tissues. Our results further show that AX coumaroylation has little or no impact on cell wall digestibility or on lignin or silicon content. This study helps clarify the roles of members of the MCBATs in cell wall biology.

## Data availability statement

The raw data supporting the conclusions of this article will be made available by the authors, without undue reservation.

## Author contributions

SM designed experiments and genetic constructs and analyzed the experimental data. CL performed the NMR analysis of the samples. NO performed the HPLC analysis of free and conjugated HCA. RS performed saccharification and monosaccharide analysis. AD identified HCAs by MS. LG and SM-M conceived and coordinated the project, experiments, and data analysis. SM, LG, and SM-M wrote the manuscript with contributions from all authors. All authors contributed to the article and approved the submitted version.

## Funding

This research was supported by funding from the BBSRC through grants BB/P022499/1 and BB/N013689/1. The York Centre of Excellence in Mass was supported by Yorkshire Forward with funds from the Northern Way Initiative, and subsequent support from EPSRC (EP/K039660/1 and EP/M028127/1). CL thanks the Leverhulme Trust Early Career Fellowship (ECF-2018-480) and the University of St Andrews.

## Conflict of interest

The authors declare that the research was conducted in the absence of any commercial or financial relationships that could be construed as a potential conflict of interest.

## Publisher’s note

All claims expressed in this article are solely those of the authors and do not necessarily represent those of their affiliated organizations, or those of the publisher, the editors and the reviewers. Any product that may be evaluated in this article, or claim that may be made by its manufacturer, is not guaranteed or endorsed by the publisher.
