# Supplementary material for: CRISPR/Cas9 suppression of OsAT10, a rice BAHD acyltransferase, reduces p-coumaric acid incorporation into arabinoxylan without increasing saccharification
Source: Front Plant Sci. 2022 Jul 22;13:926300. doi: 10.3389/fpls.2022.926300 (PMC9355400; doi:10.3389/fpls.2022.926300)
Supplement: Supplementary file 1 [file Data_Sheet_1.docx]

Supplementary Material

**Article title:** CRISPR/Cas9 suppression of OsAT10, a rice BAHD acyltransferase, reduces p-coumaric acid incorporation into arabinoxylan without increasing saccharification.

**Authors:** Svenning R. Moller^1^, Christopher S. Lancefield^2^, Nicola Oates^1^, Rachael Hallam^1^, Adam Doyle^3^, Leonardo D. Gomez^1^ and Simon J. McQueen-Mason^1^*

Corresponding author: Simon J. McQueen-Mason; [simon.mcqueenmason@york.ac.uk](mailto:simon.mcqueenmason@york.ac.uk)

**Supplementary table**

**Table 1.** *- primer list*

| Primer | Used on template (with primer) |  |
| --- | --- | --- |
| p022_crAT10.1-sg | pICSL90010 (p033) | CGTCGGGTAGCGGTCGACCC GTTTAAGAGCTATGCTGGAAACAG |
| p024_crAT10.2-sg | pICSL90010 (p033) | GACGGGGCCCTCGGACACCT GTTTAAGAGCTATGCTGGAAACAG |
| p021_U3-AT10.1 | UAP1-OsU3 (p034) | GGGTCGACCGCTACCCGACG TGCCACGGATCATCTGCAC |
| p023_U3-AT10.2 | UAP1-OsU3 (p034) | AGGTGTCCGAGGGCCCCGTC TGCCACGGATCATCTGCAC |
| p033_tracr | pICSL90010  (p022 or p024) | GCGGCCGCCAGTGGTTTAAAC ACGGCCAGTGAATTCGAG |
| P034_U3 | UAP1-OsU3  (p021 or p023) | AGCTAATTCGAGCTCGGTACCGCGGCCGC TTCGCTAAGGATGATTTCTGG |
| p010_RiceUbiR | UAP1-OsUbi (p031) | TCTTCTTCTTAGGGGCCATG CTGCAAGAAATAATCACCAAAC |
| p031_RiceUbiF | UAP1-OsUbi (p010) | GTTTAAACCACTGGCGGCCGC TTCGCTAAGGATGATTTCTGG |
| p045_(V310_zmUbiF) | V316, Hygromycin cassette (p036) | CCATCTCTTACTAGTGCCTC GTGGTCTCAGGAGGTGCAGC |
| p036_(V310_CasR) | V316, Hygromycin cassette (p045) | CTGAGACCACCTCTCG AGGATATCGCATGCTCC |
| p161 | gDNA (p162) | CGAACGGGTAGTAGTGCACC |
| p162 | gDNA (p161) | AGTCAGCCTGGTCAGTGATC |
| p181 | PCR products for sequencing | TCCTTCTTCTTCCCCTCGAC |

**Supplementary Figures:**


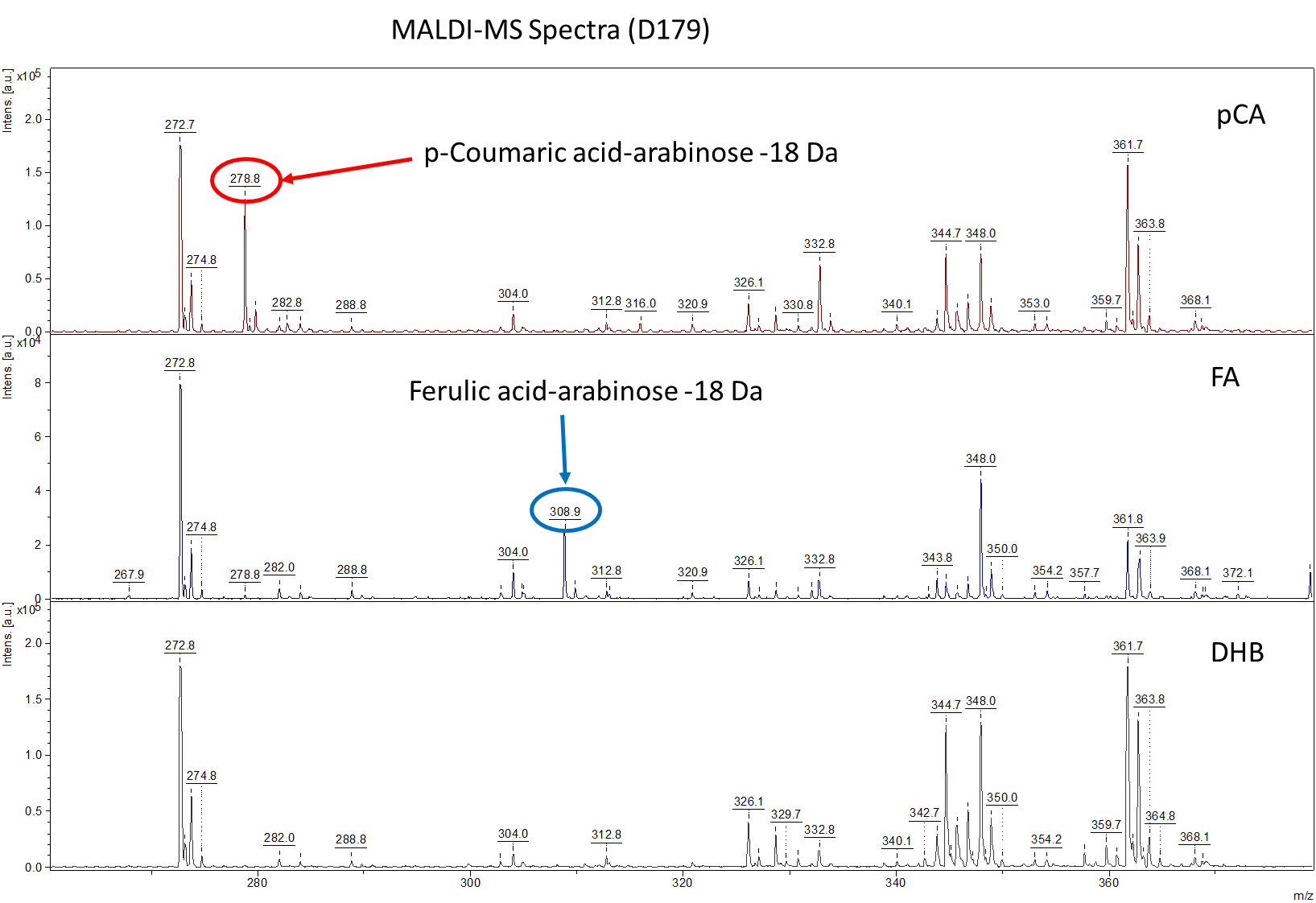


B

A


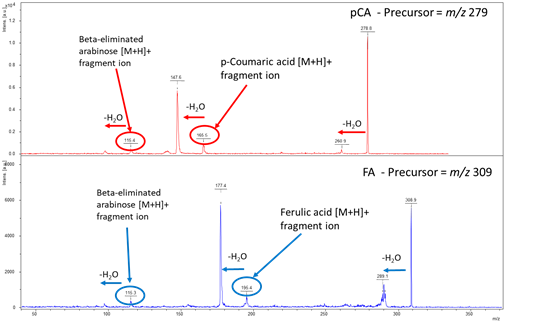


**Supplementary Figure 1.** Spectra and MALDI-TOF data*.* Acquisition was performed using a Bruker ultraflex III with positive ionisation post spotting samples 1:1 with 20 mg/ml DHB matrix. Unique signals are present in each sample at 18 Da (potentially -H_2_O) below the expected m/z values – m/z 278.8 in the p-coumaric acid-arabinose sample and m/z 308.9 in the ferulic acid-arabinose sample (**A**). To investigate the -18 Da mass observations further these two ions were selected for isolation and MALDI-MS/MS fragmentation (**B**). Loss of -18 Da (annotated as H_2_O) is common in the spectra and this is typical upon fragmentation. However, the most important features in the spectra are the ions at m/z 165 and 195 in *p*-CA and FA respectively, which are diagnostic as the protonated fragments for the expected acids, p-coumaric acid and ferulic acid. Although further water loss is seen from here, the presence of these ions indicates that in the precursor analyte the -18 Da below the expected mass must reside on the pentose. More evidence for the loss of water on the pentose in the starting material is demonstrated by the ion at m/z 115, which is the mass of the precursor [M+H]+ minus the fragmented acid component.

**Supplementary Figure 2.** Analysis of the molecular fraction (mol %) of the monomeric sugars released by TFA in the water extractable sugars fractions from OsAT10 (Hatched bars) and WT (Grey bars). **A**, Leaves. **B**, Husks.


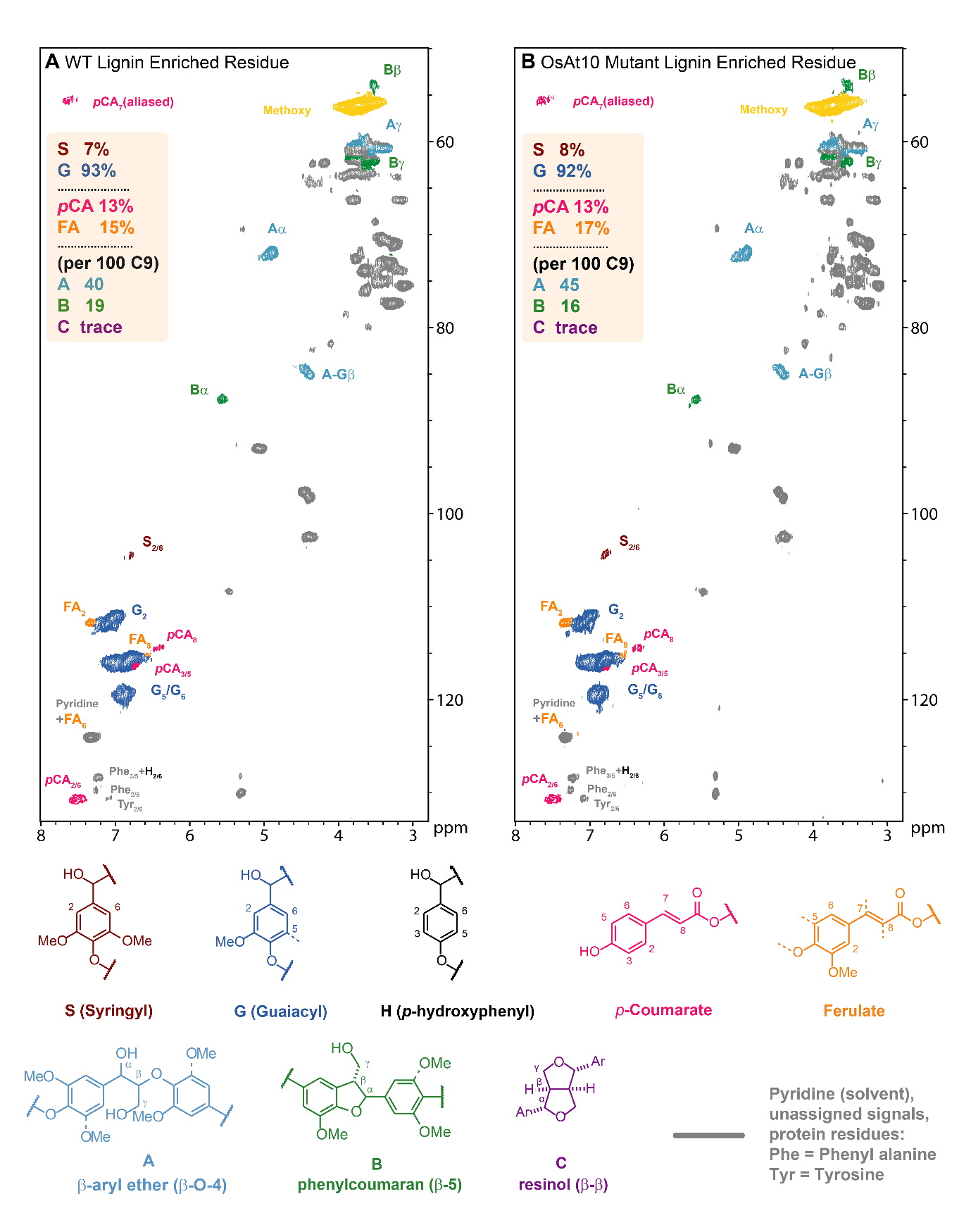


**Supplementary Figure 3.** **2D-NMR heteronuclear single-quantum coherence (HSQC) partial spectra of lignin-enriched rice husk cell wall samples**. **A,C.** shows the WT control and **B,D**. the *Osat10* knockout mutant. The analytical data are from volume integrals of correlation peaks representing reasonably well-resolved C/H pairs in similar environments; thus, they are from S2/6, G2, FA2 and *p*-CA2/6 in the aromatic region, and Aα, Bα and Cα in the linkage region, with corrections applied for units that have two C/H pairs per unit. The FA2, *p*-CA2/6 and linkage abundances are reported on a per 100 C9 unit basis relative to G + S = 100 C9 units.

*
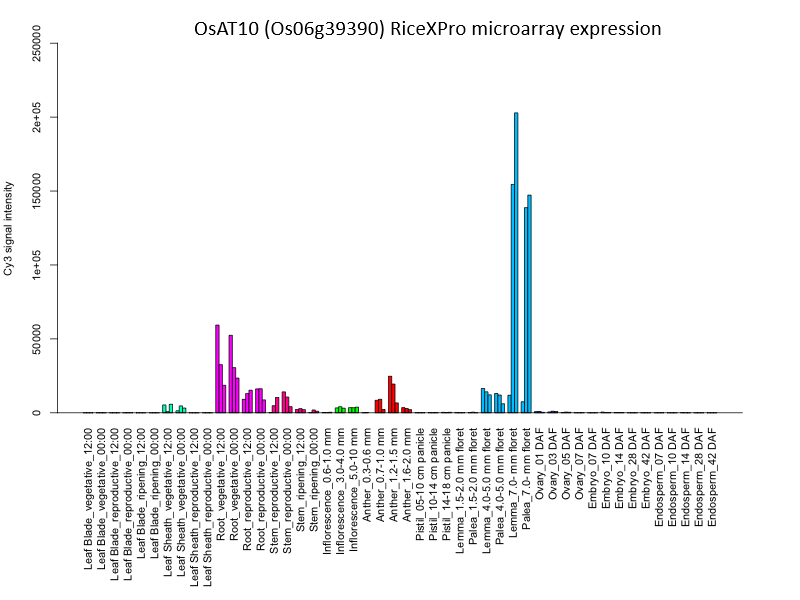
*

# Supplementary Figure 4. Expression of OsAT10 is likely mainly taking place in the lemma and palea (rice husks). Expression patterns were obtained using RiceXPro (https://ricexpro.dna.affrc.go.jp/index.html) and the dataset of [1, 2]

1. Sato, Y., et al., *RiceXPro: a platform for monitoring gene expression in japonica rice grown under natural field conditions.* Nucleic Acids Research, 2011. **39**(Database issue): p. D1141-D1148.

2. Sato, Y., et al., *RiceXPro Version 3.0: expanding the informatics resource for rice transcriptome.* Nucleic Acids Research, 2013. **41**(Database issue): p. D1206-D1213.
